# Editorial: Challenges in reaching the UNAIDS 95-95-95 targets in sub-Saharan Africa: status, innovations and pathways forward

**DOI:** 10.3389/fpubh.2025.1701425

**Published:** 2025-10-03

**Authors:** Olivier Mukuku, Kaymarlin Govender, Benard W. Kulohoma, Stanislas Okitotsho Wembonyama

**Affiliations:** ^1^Department of Research, Institut Supérieur des Techniques Médicales de Lubumbashi, Lubumbashi, Democratic Republic of Congo; ^2^Health Economics and HIV and AIDS Research Division (HEARD), University of KwaZulu-Natal, Durban, KwaZulu-Natal, South Africa; ^3^Ortholog, Public Health Research Institute, Nairobi, Kenya; ^4^Department of Pediatrics, Faculty of Medicine, University of Lubumbashi, Lubumbashi, Democratic Republic of Congo

**Keywords:** UNAIDS 95-95-95 targets, HIV testing, antiretroviral therapy, pre-exposure prophylaxis, HIV service delivery, sub-Saharan Africa

## 1 Introduction

The global commitment to ending the human immunodeficiency virus (HIV) epidemic by 2030 is anchored in the UNAIDS 95-95-95 targets: 95% of people living with HIV (PLHIV) know their status, 95% of those diagnosed receive sustained antiretroviral therapy (ART), and 95% of those on ART achieve viral suppression. While remarkable progress has been achieved in several Southern African countries, such as Botswana, Eswatini, Lesotho, Namibia, Rwanda, Zambia, and Zimbabwe, the sub-Saharan African (SSA) region continues to face formidable obstacles in the HIV care cascade. This Editorial introduces the *Research Topic* “*Challenges in reaching the UNAIDS 95-95-95 targets in sub-Saharan Africa: status, innovations and pathways forward*,” a compendium of 18 articles that collectively shed light on persistent gaps, showcase innovative approaches, and outline critical pathways to accelerate progress toward these ambitious global targets.

## 2 Status and persistent challenges

Achieving the first 95—HIV testing—remains hindered by undiagnosed infections, particularly among high-risk and marginalized populations. Several studies have highlighted the persistently low uptake of HIV testing among men, adolescents, and young women, driven by stigma, sociocultural barriers, and limited demand for services (Lancaster et al.; Govender et al.; Abebe et al.). In Tanzania, only 60% of young women reported ever being tested for HIV, with factors such as age, marital status, healthcare access, and media exposure strongly influencing uptake, while stigma and discriminatory attitudes significantly reduced testing (Lancaster et al.). In South Africa, adolescent girls and young women face psychosocial barriers, including depression, substance use, and intimate partner violence, which are associated with higher HIV vulnerability and lower engagement with testing services (Govender et al.). Similarly, in Malawi, heavy alcohol use among people seeking sexually transmitted infection (STI) care undermines HIV prevention and testing behaviors, underscoring the need for integrated, status-neutral interventions that address both substance use and HIV testing (Abebe et al.). These findings emphasize that multifaceted, context-specific strategies are essential for improving testing coverage and accelerating progress toward the first 95.

Children are disproportionately affected, lagging behind adults in terms of diagnosis and treatment initiation, thereby threatening the timely attainment of treatment targets (Kulohoma and Wesonga (a)). First, children lag behind adults in diagnosis, treatment uptake, and viral suppression, which threatens the timely achievement of UNAIDS targets. For instance, only 67% of HIV-exposed infants were tested within 2 months of birth in 2023, and only 29% of children under 15 years initiated ART before their 5th birthday, compared to higher coverage rates among adults (Kulohoma and Wesonga (b)). Therefore, tailored interventions for children are crucial to avoid delays in their overall progress. Second, there is a loss to follow-up in people living with HIV receiving ART, especially in rural settings, where reliance on paper-based records is a key challenge. Geographic disparities further exacerbate inequities, as shown in Cameroon, where HIV prevalence declined from 6.9% to 2.7% over a decade in one region but stagnated at ~5% in another region, underscoring how underserved areas frequently experience delayed diagnosis due to insufficient service availability (Ngoume et al.).

The second 95—linkage to care and retention—faces challenges such as prompt ART initiation, continuous engagement in care, and monitoring. High rates of loss to follow-up have been reported in rural settings, often compounded by the reliance on paper-based medical records (Abugah et al.; Amhare et al.). Structural barriers such as transportation constraints, health system limitations, and socioeconomic hardships, hinder consistent care. Similar implementation challenges are evident in Ethiopia, where an evaluation of the ART program at Woldia General Hospital reported an overall program implementation of 74.8%, healthcare personnel adherence to national guidelines of only 66%, and patient satisfaction below the national targets (Seid et al.). Shortages of test kits, essential medications, and inconsistent laboratory testing further exacerbate these challenges. While innovations in patient tracking and the digitisation of health records have shown promise for improving retention, scaling these solutions remains inconsistent (Abugah et al.).

The third 95—viral suppression—is intricately linked to treatment adherence, regimen optimisation, and monitoring capacity. Viral rebound is frequently associated with adherence challenges, drug resistance, limited viral load monitoring, and regimen complexity (Boateng et al.; Dzomba et al.). Recent data from Ghana showed that although 74% of patients achieved viral suppression at baseline, this rate increased to 88% after 18 months. However, viral rebound persisted at 13.6 per 1,000 person-months, particularly among individuals with lower education levels (Boateng et al.). In rural South Africa, sustained suppression reached up to 88% between 2017 and 2019; however, 2–3% of previously suppressed individuals experienced rebound annually, and ~5% had treatment failure (Dzomba et al.). In Ethiopia, an evaluation of the ART program revealed substantial implementation gaps, with guideline adherence among health workers at only 66%, and persistent shortages of viral load testing kits, further undermining viral suppression efforts (Seid et al.). Socioeconomic hardship, mental health concerns, and stigma further exacerbate these issues, underscoring the need for integrated, and patient-centered approaches.

Financial constraints are pervasive obstacles across the cascade. Insufficient domestic and international funding threatens both the sustainability of HIV programs and the scaling up of innovative interventions (Kulohoma and Wesonga (a); Kulohoma and Wesonga (b)). Financial barriers impede progress toward the UNAIDS 95-95-95 HIV targets by constraining the availability and scalability of HIV testing, treatment access, and adherence support, leading to poor viral suppression in SSA. Innovative financing mechanisms, improved resource mobilization strategies, and coordinated efforts to ensure long-term sustainability and closer alignment with global HIV goals are necessary to bridge these funding gaps. Gaps in political commitment, resource mobilization, and coordinated planning risk derailing progress in achieving the 95-95-95 targets across the region.

## 3 Innovations and strategies for progress

Despite these persistent challenges, recent studies have highlighted effective innovations in HIV service delivery. Index testing, community-based outreach, and HIV self-testing have increased case detection among previously undiagnosed individuals (Lancaster et al.; Moraka et al.). In Botswana, up to 30% of ART initiators had undisclosed prior antiretroviral use, emphasizing the need for pre-treatment drug screening (Moraka et al.). In Malawi, a status-neutral intervention with alcohol-reduction counseling in STI clinics improved prevention and treatment uptake among heavy drinkers (Lancaster et al.). Targeted strategies have also enhanced HIV testing in SSA: 60% of young women in Tanzania reported ever being tested in 2022 (Abebe et al.), while in Guinea, where the HIV prevalence among men who have sex with men reached 9.4% in 2022, stigma-free tailored interventions are urgently required for timely diagnosis (Dioum et al.).

The decentralization of ART services, nurse-led clinics, and task-shifting initiatives have facilitated access to underserved regions, while community engagement and peer-support interventions have strengthened retention and adherence (Govender et al.; Omotoso et al.). Quality improvement initiatives at clinics, integration of partner notification, and geospatial mapping for localized interventions have proven effective in addressing geographic disparities and improving viral suppression outcomes (Fwemba et al.; Lancaster et al.).

Emerging biomedical prevention tools, such as long-acting injectable pre-exposure prophylaxis (PrEP), show promise in reducing the incidence of new HIV infections. However, access and affordability remain challenges, particularly in resource-constrained settings (Kileku et al.; Shangase et al.). Pediatric ART registries and implementation science approaches have demonstrated that structured, data-driven monitoring of pediatric populations can accelerate progress in reaching the first and second 95 of children (Omotoso et al., Magura et al.).

## 4 Pathways forward

To bridge the remaining gaps, sustained political will and financial commitment, both domestic and international, are indispensable. Innovative financing mechanisms, enhanced resource mobilization, and strengthened coordination among stakeholders are vital for ensuring long-term sustainability (Kulohoma and Wesonga (a); Kulohoma and Wesonga (b)). Community-led approaches that reduce stigma, address structural barriers, and engage local actors are essential for reaching hard-to-reach populations (Govender et al.; Dioum et al.).

Digital health innovations, including electronic patient records, mobile health reminders, and geospatially informed interventions, offer promising avenues for improving retention and adherence to treatment. Digitizing health records, enhancing patient-tracking systems, and adopting community engagement strategies can improve retention. Socioeconomic hardship, stigma, health system limitations, and individual and structural challenges undermine progress. Treatment adherence, regimen type, monitoring frequency, and patient demographics influenced the viral load outcomes. Robust viral load monitoring, patient adherence support, and regimen monitoring and optimisation are crucial. Targeted public health interventions, including educational campaigns and improved access, enhance HIV testing uptake among young women. Multifaceted interventions that strengthen healthcare delivery, reduce stigma, integrate mental health and social support, and enhance patient-centered care approaches can improve adherence and accelerate target achievement. Evidence from Ethiopia and South Africa suggests that combining digital tools with community engagement enhances care continuity and patient-centered support (Amhare et al.; Dzomba et al.). Multifaceted interventions integrating mental health and social support are equally vital, considering their impact on adherence and on viral suppression.

Intensified prevention and treatment strategies tailored to the local epidemiological context are essential. Targeted educational campaigns, PrEP scale-up, and the integration of behavioral interventions, such as alcohol reduction programs in STI care settings, address intersectional vulnerabilities and enhance engagement with care (Lancaster et al.; Kileku et al.). Pediatric-focused interventions, including early diagnosis, ART initiation, and registry-based monitoring, are necessary to close age-related gaps in progress (Omotoso et al.; Magura et al.).

The adoption of emerging technologies must be accompanied by deliberate strategies to ensure equitable access. Access to long-acting ART, viral load monitoring, and PrEP must be expanded to rural and marginalized populations to avoid widening the existing disparities. Data-driven approaches that combine routine program data, geospatial analytics, and implementation science frameworks can guide resource allocation and inform adaptive strategies that respond to local needs (Fwemba et al.; Magura et al.).

## 5 Conclusion

The path to achieving the UNAIDS 95-95-95 targets in SSA is complex and shaped by interlinked challenges spanning HIV testing, treatment, and viral suppression. Insights from this Research Topic underscore that progress requires a multifaceted response: innovative biomedical, behavioral, and structural interventions; strengthened health systems; robust financing; and increased community engagement. Lessons from successful countries provide a roadmap; however, scaling up effective strategies across diverse contexts remains a critical challenge.

By integrating evidence-based innovations, leveraging digital technologies, and promoting equitable access to care, SSA can accelerate progress toward 95-95-95, moving closer to ending the HIV epidemic by 2030. The collective findings from these 18 studies illuminate both the obstacles and transformative potential of targeted and context-specific interventions. With sustained commitment, collaboration, and innovation, the UNAIDS targets can serve as aspirational goals and achievable milestones in the global fight against HIV.

